# HIV and sexual risk behaviours by 18-25-year-old youth at Nyandeni Municipality in the Eastern Cape

**DOI:** 10.4102/hsag.v29i0.2541

**Published:** 2024-08-30

**Authors:** Lorraine N. Mntonintshi-Mketo, Robert T. Netangaheni, Moganki H. Lefoka

**Affiliations:** 1 Department of Health Studies, Faculty of Human Sciences, University of South Africa, Pretoria, South Africa; 2 Department of Sociology, Faculty of Human Sciences, University of South Africa, Pretoria, South Africa; 3 Department of Family Medicine, School of Medicine, Faculty of Health Sciences, University of Pretoria, Pretoria, South Africa

**Keywords:** HIV, sexual risk, behaviours, knowledge, attitudes, youth

## Abstract

**Background:**

The human immunodeficiency virus (HIV) epidemic in South Africa is among the worst in the world; in 2017, 38% of new infections were among young people aged 15–24 years. Estimates for HIV infection in 2020 worldwide indicate that there will be 1.5 million new cases, 10.2 million untreated cases (out of 37.7 million), and 680 000 deaths from acquired immunodeficiency syndrome (AIDS). Despite a 46% decline in new HIV infections among adolescents and youth over the previous 10 years, two of the seven new HIV infections in 2019 occurred in people between the ages of 15 and 24. HIV prevalence among young people has remained unchanged since 2008. This consistent pattern among people under 30 years of age indicates a failure in HIV prevention.

**Aim:**

The study aimed to explore HIV and sexual risk behaviours by 18–25-year-old youth at Nyandeni Municipality in the Eastern Cape province.

**Setting:**

The investigation was conducted Nyandeni Municipality in the Eastern Cape province.

**Methods:**

Qualitative approach was used to explore, describe and investigate the knowledge and attitudes about HIV among the 18–25 years old youth

**Results:**

The findings are based on three themes namely, knowledge and attitudes about HIV and AIDS in youth, sexual risk behaviour among youth, and HIV prevention strategies.

**Conclusion:**

This exploratory investigation confirms that the participants’ knowledge is limited by showing that most of them knew very little about HIV and AIDS infection and prevention. Ongoing educational initiatives are required.

**Contribution:**

Youth experience high HIV incidence because of their knowledge gaps.

## Introduction

South Africa has one of the world’s largest human immunodeficiency virus (HIV) epidemics, with youth aged 15–24 years accounting for 38% of new infections in 2017 (Pleaner et al. 2023). Estimates for HIV infection worldwide in 2020 indicate that there will be 1.5 million new cases, 10.2 million untreated cases (out of 37.7 million), and 680 000 deaths from acquired immunodeficiency syndrome (AIDS). The HIV infection is still a public health concern. Despite a 46% decline in new HIV infections among adolescents and youth over the previous 10 years, two of the seven new HIV infections occurred in people between the ages of 15 years and 24 years (Bossonario et al. 2022). Global data indicate that 5.1 million adolescents and young people were living with HIV in 2019.

Sub-Saharan Africa (SSA) is home to 71% of HIV-positive individuals and 65% of newly diagnosed cases in 2017 (Khamisa, Mokgobi & Basera 2020). This region bears the brunt of the HIV burden. The fact that approximately 2 million young people living with HIV/AIDS reside in SSA, an area disproportionately impacted by the infection, is equally concerning. Growing data show that young people with HIV are more likely to die from HIV-related causes, have lower rates of adherence and viral suppression, and are gradually kept out of care (Woollett, Pahad & Black 2021). According to the UNAIDS 2016 global report, 20% of new HIV infections in SSA occur in young people between the ages of 15 years and 24 years, (Chetty-Makkan et al. 2020). The Nyandeni Local Municipality Integrated Development Plan of 2015/2016 reported 50% – 60% of new infections among youths aged 15–30 years in 2014 (Nyandeni Integrated Development Plan 2018).

Youths are disproportionately affected by HIV. According to Hamid et al. (2022), youth is described as the time between childhood and adulthood when young people are most susceptible to high-risk behaviours, especially those related to sexuality. A lack of information regarding HIV transmission and prevention has been recognised as one of the risk factors for the epidemic’s spread among young people. In addition to not knowing enough about HIV, other risk factors for HIV infection in young people include using dating apps, using illicit drugs, drinking alcohol before having sex, using multiple partners, and not using condoms during sexual interactions (Lima et al. 2020)

According to Chetty-Makkan et al. (2020), the prevalence of HIV among youth in South Africa (15–24 years) is still high. In 2017, the country’s youths had an 8% HIV prevalence rate, with 5% prevalence among males and 11% prevalence among females. Adolescent girls and young women (AGYW) in South Africa bear a disproportionate burden of HIV. This is particularly noticeable in the 15–19 age group (4.7% males vs. 5.8% females) and even more so in the 20–24 age group (4.8% males vs. 15.6% females) (Abdullha et al. 2019). Cross-cutting variables such as age disparate sex, transactional sex, poverty, and unemployment, combined with negative gender norms and unequal gender power dynamics, seriously jeopardise the rights, safety, and choices of adolescent girls and women (Mullick 2023; Pleaner et al. 2023). The greatest HIV epidemic in the world is currently occurring in South Africa, where AGYW between the ages of 15 and 24 account for 25% of all new infections (Abdullah et al. 2019).

An estimated 7.92 million South Africans of all ages are expected to be HIV positive, according to the country’s 14% HIV prevalence estimate. Numerous biological, socio-behavioural, environmental, and structural factors have been implicated in the high prevalence of HIV in South Africa. According to Zuma et al. (2022), most HIV infections in South Africa are caused by heterosexual transmission. The nation has made great strides in recent years to assist more South Africans who are HIV positive in getting access to treatment. Because antiretroviral therapy (ART) reduces mortality and morbidity and lengthens longevity, it has enhanced the quality of life for persons living with HIV (Varshney et al. 2022).

Important results from the 2017 National Population-Based Survey conducted in South Africa reveal that a significant number of people are still contracting HIV, especially youth, where the prevalence of the virus has not altered since 2008. According to Zuma et al. (2022), this pattern of constancy in individuals under 30 years of age suggests a failure in HIV prevention. Young males who do not test for HIV add to the difficulties in meeting the first 90 (HIV testing) of UNAIDS’ 90-90-90 targets for South Africa, even though the disease primarily affects women (Shamu et al. 2020).

The poor health outcomes of adolescents living with HIV have been the subject of recent systematic reviews that have attempted to identify the contributing causes. Reasons for this include stigma, the perception of a lack of confidentiality at clinics, treatment fatigue, inadequate care from healthcare professionals, ineffective transitions from paediatric to adult clinics, inadequate disclosure, and a dearth of services geared towards adolescents (Woollett et al. 2021). The percentage of South African youth who have had HIV testing is only approximately 67%, despite their high risk of contracting the virus. Some of the most frequent obstacles to HIV testing among young people are a lack of understanding about the virus, concerns about confidentiality being breached or receiving a HIV testing uptake is still insufficient despite national attempts to get youths involved in HIV testing services (HTS) through youth-friendly health services (Chetty-Makkan et al. 2020).

Understanding HIV and sexually transmitted infections (STIs) is necessary to improve youth’s ability to engage in safe sexual conduct and to address negative attitudes towards the use of condoms. In order to address the increased prevalence of HIV and STIs in vulnerable populations, this is probably going to lead to a greater adoption of HIV prevention techniques. Young women are more likely than young men to be infected with HIV and AIDS, and unprotected sex is the most prevalent way for the infections to spread. Negative attitudes, risk sexual conduct, and a lack of awareness are the main obstacles to prevention (Khamisa et al. 2020).

The lives of persons who contract HIV are profoundly affected by the virus. Mental health problems are common among young individuals living with HIV, and they can negatively affect adherence and retention in care, among other things. Worldwide, HIV-positive youth’s mental health needs are grossly neglected in low- and middle-income nations and do not receive the resources and care they need (Woollett et al. 2021). This figure is concerning because it shows that we are still a long way from meeting the new 95-95-95 target, and it will put all HIV-related initiatives in South Africa at risk. This study aims to investigate HIV and sexual risk behaviours by 18–25-year-old youth at Nyandeni Municipality in the Eastern Cape province in order to better understand the youth’s HIV knowledge and attitudes, as well as to suggest HIV prevention strategies.

### Aim of the study

In order to identify the youth’s current level of knowledge and risk sexual behaviour, the study set out to explore the attitudes, knowledge, and risk sexual behaviour of youth in the Nyandeni Municipality in the Eastern Cape province. It also made recommendations for a future education programme as a preventive measure.

## Research methods and design

### Study design

A research design is defined as a set of formal procedures for gathering, analysing, and interpreting data (Creswell & Creswell 2018). The research design guides how the study will unfold and further allows research questions to be answered and research objectives to be met (Kumar 2018:41). Exploratory and descriptive research designs were used to explore and describe the study purpose. The choice of research design and data collection method that were utilised in this study was based on the research objectives intended at understanding the level of knowledge about HIV and AIDS.

### Setting

The study was conducted in Nyandeni Municipality, one of the smallest local municipality in the OR Tambo District in the Eastern Cape. The participants were recruited at two non-profit organisations (NPOs), namely at Philisa Home Based Care Centre and Nompilo M U Home. The NPOs were selected because they provide HIV-related services to young people. It has been reported that among tested cases of young men and women between the ages of 15 and 30, the occurrence rate of HIV and AIDS was as high as 50% – 60% in 2014. The majority of these instances involved men who came to the nearby clinics with symptoms of sexually transmitted diseases (STDs), and women who took part in voluntary testing during routine prenatal appointments (Nyandeni Integrated Development Plan 2018).

### Study population and sampling strategy

The population of this study were youths between the ages of 18 and 25 residing in Nyandeni Municipality in the Eastern Cape province. Both males and females were considered for the study. Inclusion and exclusion criteria were followed during the sampling process. The findings of the census (Statistics SA, 2022) revealed that young people aged 15–29 years make up a quarter of the population of Nyandeni, which is 25.5%.

Convenience sampling and non-probability purposive sampling methods were used to choose the study sample. For this research study, a non-probability sampling technique was used. The researcher recruited and sampled the participants at Philisa Home Based Care and Nompilo M U Home. Because of time and financial constraints, the researcher recruited 30 participants from 162 NPO beneficiaries from both the NPOs. A total of 15 participants were selected from 89 beneficiaries of Philisa Home Based Care Centre and another 15 participants were selected from 73 beneficiaries of Nompilo M U Home.

The following inclusion and exclusion criteria were followed:

In Inclusion criteria:

- Youths, male and female aged between 18 and 25 and irrespective of race
- Resident of (name deleted to maintain the integrity of the review process)
- Youths with intact or uncompromised mental capacity
- Be able to give consent for participating in the study
- Youths living in rural or urban areas of (name deleted to maintain the integrity of the review process)

Exclusion criteria were as follows:

- Youths below 18 years and above 25 years of age
- None resident of (name deleted to maintain the integrity of the review process)
- Youths not meeting the above inclusion criteria

The researcher verified with the participants if they understood the purpose of the research before collecting data. The risks, benefits and their rights were discussed at length with the participants before they consented to participate in the study. All participants were allowed an opportunity to accept or decline to participate in the research study. Prior to beginning a semi-structured interview, researchers gave participants an explanation of the study’s purpose and got their signed informed consent forms.

### Data collection methods and procedures

In this study, the researcher utilised semi-structured interviews in addition to the collection of secondary data through the literature review process. The interview guide was prepared on time in order to control the interview session by allowing the participants to elaborate on their responses to the interview questions. A general central question, ‘what is your understanding of the difference between HIV and AIDS?’ served as the starting point for all of the interviews.

### Data collection

To determine whether the interview schedule could obtain the necessary data from the participants, a pilot study was carried out prior to the real data collection. Three study participants who satisfied the requirements for sample inclusion participated in the pilot investigation. Following that, two people were not included in the research study. The researcher collected data from 30 participants using in-depth semi-structured interviews. The researcher first collected data from 15 participants at Philisa Home Based Care Centre, who were interviewed between the 05th and 08th of August 2019. Additional 15 participants from Nompilo M U Home were interviewed between 12th and 16th of August 2019. An in-depth individual interview was conducted through the aid of an interview guide. The researcher used qualitative observation, described as an involvement of taking field notes on the behaviour and non-verbal communication of the participants. However, no data saturation was reached. There were no challenges encountered during data collection.

All transcriptions (audio records, note pad notes, copies of consent forms) were anonymised before analysis. A tape recorder was used by the researcher to make sure that she did not omit any information given by the interviewee. Before the interview session began, the interviewee was provided with a consent form to sign. Before signing the consent form, the researcher did explain everything to the interviewee using the language the interviewee understood. This means that the interview session was performed in the interviewee’s home language.

### Data analysis

Data were analysed thematically using six phases as identified by Braun and Clarks (Jense & Laurie 2016).

*Phase 1:* The researchers acquainted themselves with the data.

The audio recordings of the 30 interviews were verbatim transcribed by the researchers. The researchers were able to familiarise themselves with the content of the transcripts through the transcription process.

*Phase 2:* The researcher generated initial codes.

The researchers went through the transcripts one by one, critically identifying initial codes, allowing them to code a large number of codes that could form different themes.

*Phase 3:* The researcher searched for themes.

The researchers began by grouping together codes that were related to one another into possible themes. Finally, the researchers used a tabular form to neatly organise the codes and themes.

*Phase 4:* The researcher reviewed themes.

All of the themes were reviewed by the researchers. Sub-themes were created to accommodate themes that are complementary.

*Phase 5:* The researcher defined and named the themes.

This phase began when the researchers were satisfied with the themes and sub-themes identified. It was determined what each theme was about and what type of data each theme captured.

*Phase 6:* The researcher produced the report.

The researchers began writing up the data’s thematic analysis. This provided a succinct, coherent, logical, non-repetitive and interesting account of the knowledge and attitudes about HIV among the 18–25 years old youth residing in the Nyandeni Municipality in the Eastern Cape province.

### Ethical considerations

Ethical approval to conduct this study was obtained from the University of South Africa, College of Human Science Research Ethics Committee (REC-240816-052).

This study was guided by social science ethical consideration and measures to ensure trustworthiness, which were followed to the core. Furthermore, the study was approved by the Research Ethics Committee of the College of Human Science, University of South Africa. Permission was granted before data were collected. The researcher was granted permission by Department of Social Development, Philisa Home Based Care Centre and Nompilo M U Home. To protect participants’ identity and information, the researcher created a file using her computer, which is protected by a password. The researcher will keep the data for a period of 5 years, thereafter it will be permanently erased.

### Measures to ensure trustworthiness

#### Credibility

True, credible, and believable research outcomes are those that the research audience will not have cause to doubt (Korstjens & Moser 2018). In addition, a study needs to include participants who can respond to research questions in order to be considered credible (Kyngäs, Kääriäinen & Elo 2019). By developing a rapport with the participants, the researcher increased their trustworthiness. To do this, the researcher took the time to visit the NPOs where the data would be collected, gave the participants an explanation of the study, and answered any questions they had.

#### Transferability

The degree to which the findings of qualitative research can be extrapolated or used to different situations is known as transferability (Kumar 2018). The researcher gave careful thought to filed notes, observation, and examination of non-verbal clues during the interviews.

#### Confirmability

The degree to which research findings may be independently verified as true is known as confirmability (Forero et al. 2018; Korstjens & Moser 2018). Confirmability of a study’s findings and conclusions is obtained when the data gathered can support them. By audio recording the participants, checking in with them, asking follow-up questions, and not presuming to know what the researcher does not comprehend, confirmability was obtained. Additionally, the researcher made sure that her views and ideas about the subject were kept private.

#### Dependability

According to Nieswiadomy and Bailey (2018), dependability is a subject of research audit that necessitates an auditor to evaluate and scrutinise the research process and its results. Gray and Grove (2020) state that ‘documentation of steps taken, and decisions made during analysis’ is a key component of dependability. The researcher detailed every step and technique used in the research methodology in order to guarantee reliability. Furthermore, in order to guarantee reliability, the second author – a skilled qualitative and mix-method researcher – helped with co-coding.

## Results

Data were collected from a sample of thirty participants (see Table 1). Three main themes and 12 sub-themes emerged from the data analysis (see Table 2).

**TABLE 1:** Biographical characteristics of the participants.

Participant	Age (years)	Gender	Marital Status	Grade	Urban or Rural	Home language	Employment status
1	20	M	Single	8	Rural	isiXhosa	Employed
2	24	F	widowed	11	Rural	isiXhosa	Not employed
3	25	M	Single	6	Rural	isiXhosa	Not employed
4	20	F	Single	10	Rural	isiXhosa	Employed
5	18	M	Single	11	Rural	isiXhosa	Not employed
6	24	F	Single	7	Rural	isiXhosa	Employed
7	25	M	Single	Tertiary	Urban	isiXhosa	Not employed
8	19	F	Single	9	Rural	isiXhosa	Not employed
9	25	F	Married	12	Rural	isiXhosa	Employed
10	25	M	Single	6	Rural	isiXhosa	Not employed
11	18	F	Single	11	Rural	isiXhosa	Not employed
12	19	M	Single	12	Rural	isiXhosa	Employed
13	21	F	Single	Tertiary	Rural	isiZulu	Not employed
14	20	F	Single	12	Rural	isiXhosa	Employed
15	18	M	Single	10	Urban	isiXhosa	Not employed
16	22	M	Single	10	Rural	isiXhosa	Employed
17	21	F	Single	12	Rural	isiXhosa	Not employed
18	24	F	Divorced	11	Rural	isiXhosa	Not employed
19	21	M	Single	12	Rural	isiXhosa	Employed
20	22	M	Single	10	Rural	isiXhosa	Not employed
21	19	F	Single	11	Rural	isiXhosa	Employed
22	24	F	Divorced	10	Rural	isiXhosa	Employed
23	23	M	Single	Tertiary	Urban	isiXhosa	Not employed
24	19	M	Single	10	Rural	isiXhosa	Not employed
25	18	F	Single	10	Rural	isiXhosa	Not employed
26	22	M	Single	12	Rural	isiXhosa	Employed
27	20	F	Single	Tertiary	Rural	isiXhosa	Employed
28	21	M	Single	10	Rural	isiXhosa	Employed
29	25	M	Single	Tertiary	Rural	isiXhosa	Not employed
30	21	M	Single	11	Rural	isiXhosa	Employed

Female, female; M, male.

**TABLE 2:** Themes and sub-themes.

Themes		Sub-themes
1.	HIV and AIDS knowledge and attitudes in youth	1.1. Modes of HIV transmission 1.2. Perceptions regarding HIV and AIDS 1.3. Fear of disclosing HIV status 1.4. Perception on HIV infection 1.5. Attitude towards testing HIV positive 1.6. Perceptions about condom use
2.	Sexual risk behaviour among youth	2.1. Perceived risk of contracting HIV 2.2. Sexual risk understanding 2.3. Factors associated with sexual risk behaviour
3.	HIV prevention strategies	3.1. HIV education awareness programmes 3.2. Distribution of condom and enrolment on pre-exposure prophylaxis (PrEP) 3.3. HIV testing services

### Theme 1: HIV and AIDS knowledge and attitudes in youth

One participant, aged 21, stated unequivocally that they do not know the difference between HIV and AIDS, which is problematic. Participants reported that:

‘HIV and AIDS are almost the same, when you have HIV you cough non-stop but with AIDS it is when your organs are badly damaged.’ (Participant 3, Male, 25 years old)

‘I know AIDS kills and you lose weight, the same goes for HIV, there is no difference because you die in the end.’ (Participant 8, Female, 19 years old)

‘HIV is a virus; AIDS is the same as the virus.’ (Participant 11, Female, 18 years old)

‘AIDS is a disease whereas HIV is a virus from the infected blood.’ (Participant 17, Female, 21 years old)

‘I do not know the difference; I think they are the same.’ (Participant 30, Male, 21 years)

#### Sub-theme 1.1: Modes of HIV transmission

Most participants reported that HIV is transferred through contact with infected blood when there is cut and that blood comes into contact with infected blood. Two participants reported that HIV can be spread vertically. Other participants reported that HIV can be conveyed by sharing a spoon, from spitted septum which evaporates in the air and has been inhaled by others, who then become HIV positive reflecting a HIV knowledge gap. Participants reported that:

‘A little, but I have enough information to avoid being infected by AIDS. Having sex without a condom, when you have a cut and it gets into contact with infected blood, drinking from a cup used by an HIV infected person, if his or her lips have sores.’ (Participant 1, Male, 20 years old)

‘I have enough knowledge to look after myself not using protection like a condom, not using gloves when helping in an accident, using other people’s toothbrush to brush your teeth.’ (Participant 4, Female, 20 years old)

‘I cannot be sure how much know because every day there is something new to know about HIV unprotected sex, and when you have a cut and you touch infected blood.’ (Participant 5, Male, 18 years)

#### Sub-theme 1.2: Perceptions regarding HIV and AIDS

Participants recognise the importance of seeking treatment after being diagnosed with HIV. Although one participant stated that if a person develops sores in the private area, they must consult a traditional healer, and another participant stated that HIV is curable but has not yet been confirmed to the public, the participants support HIV treatment in assisting one to enjoy quality of health. Participants reported that:

‘There must be counselling before and after testing so that the person does not get depressed when tested positive, the disease affects all people. Once you test positive you must disclose to a person you trust for relief and then eat your treatment and green vegetables not forgetting lots of fruit. Take treatment at the chosen fixed time to avoid defaulting.’ (Participant 24, Male, 19 years old)

‘Government has provided early taking of treatment called Universal Test and Treat [*UTT*]. The more you eat your treatment the more the viral load lowers and the CD4 count stays high. Eating the treatment when you test positive is a priority now. People hide their HIV status fearing of discrimination especially when the person was promiscuous.’ (Participant 25, Male, 19 years old)

‘You can live a long-time being HIV positive as long as you are taking treatment and follow the clinic guidelines.’ (Participant 26, Male, 22 years old)

‘Eating treatment on time is crucial, avoid defaulting when on treatment, lower alcohol intake and smoking.’ (Participant 27, Female, 20 years old)

‘HIV is incurable and can be suppressed by ARV pills.’ (Participant 8, Female, 19 years old)

‘Once you have HIV you develop sores in your private parts, then it destroys your insides like livers and lungs, there are constant discharges’. These sores can be removed by a traditional healer [*Igqirha*] if seen early. If the healer cannot help, the person can try western medicine [*uGqirha*] (Participant 28, Male, 20 years old)

‘HIV is a disease, there are research done but not yet released to the public, it is curable and not yet confirmed to public. If you eat pills you can lead a normal life and accepting it is no.1.’ (Participant 10, Male, 25 years old)

#### Sub-theme 1.3: Fear of disclosing HIV status

Participants associate HIV with promiscuity, which is based on stigma that prevents people from accepting their HIV status. Those who are affected are afraid of coming out because they will be told they were looking for it, so they do not seek treatment and allow their health to deteriorate without seeking medical attention. Participants reported that:

‘HIV is much in our area, many people especially youth hide it and you would see a person getting thinner day by day, they do not want to talk about it as if they will be known they are positive. Some youth in my community fear they will be reminded of their promiscuous behaviours and be told they deserve the HIV and were looking for it.’ (Participant 29, Male, 25 years old)

‘AIDS and HIV are not ok; AIDS can stop if you look after yourself’. Sometimes sleeping around like a prostitute for financial gain can result in being transmitted with it.’ (Participant 30, Male, 21 years)

#### Sub-theme 1.4: Perception on HIV infection

Participants’ attitudes towards HIV vary; some are fearful, while others believe it is a deadly virus, while others are optimistic and recognise that if they become infected, it is not the end of their lives because clinical guidelines are in place to help those who are infected. Participants reported that:

‘I am afraid of HIV, it is not curable, and it can be suppressed by ARVs.’ (Participant 1, Male, 20 years old)

‘HIV kills but it takes long to kill you, a member from my family had HIV and was eating treatment from the clinic, though he died by car accident.’ (Participant 2, Female, 24 years old)

‘The word itself makes smells like death. Once you hear someone you know is HIV positive you think of him [*or*] her dying soon.’ (Participant 20, Male, 22 years old)

‘If test HIV positive it is not the end of life, when you test positive follow the clinic guidelines for survival.’ (Participant 22, Female, 24 years old)

‘HIV does not kill, it is a better disease unlike those which kill you unexpectedly like high blood pressure, Diabetics. If you test positive, it is important to check viral load and CD4 count once in a while.’ (Participant 23, Male, 23 years old)

#### Sub-theme 1.5: Attitude towards testing HIV positive

Participants express that if they were to test HIV positive, they will feel conflicted. Some participants expressed concern, while others said they would be tortured and hurt, but they all agreed that being HIV positive would change their lives. Others went on to say that it will be difficult to disclose their HIV status and that they may withdraw. Participants reported that:

‘I would be very worried of people gossiping about me and my status, but I would learn to ignore and eating [*take*] treatment as soon as I test positive.’ (Participant 2, Female, 24 years old)

‘It would torture me a great deal but finally I will have to accept and move on.’ (Participant 7, Male, 25 years old)

‘I would be hurt, finally comes to terms with it but that would not stop me from drinking alcohol and smoking with friends because that how I chill and enjoy myself.’ (Participant 8, Female, 19 years old)

‘Testing HIV positive would change me a lot, because now I would have to eat pills every day and I really hate pills I prefer liquid medicine.’ (Participant 9, Female, 19 years old)

‘My life would never be the same again, I would be lonely. I don’t who would I trust to disclose my status situation.’ (Participant 29, Male, 25 years old)

#### Sub-theme 1.6: Perceptions about condom use

The majority of participants agreed that using a condom is critical because it not only prevents STIs and HIV but also unwanted pregnancies. Other participants stated that condoms can help to prevent the spread of HIV. Some participants, on the other hand, responded that condoms alone cannot prevent the spread of HIV, citing various reasons such as HIV not being transmitted solely through sex and that there are numerous other ways to get HIV. Participants reported that HIV is transmittable in various ways and that most people do not want to use condoms. However, one participant expressed doubt that it can be reduced solely by condoms. Participants reported that:

‘Condom plays a very important role in lives, it protects us from dreadful diseases, and it also helps in preventing pregnancies. Yes, it can reduce the spread of HIV.’ (Participant 1, Male, 20 years old)

‘Condom is vital to us youth, in these days children have children, we cheat all the time. Yes.’ (Participant 4, Female, 20 years old)

‘Yes, if all people could use condoms, Department of Health to place condoms in taverns and community shops for easy access, following that they should provide teaching on the right ways of wearing condoms to avoid breakings.’ (Participant 17, Female, 21 years old)

‘I prefer to wear a condom because I am too young to die and to have a child, trusting someone is hard in these days. Yes, I believe it can be reduced.’ (Participant 29, Male, 25 years old)

### Theme 2: Sexual risk behaviour among youth

#### Sub-theme 2.1: Perceived risk of contracting HIV

Participants were divided on the perceived risk of contracting HIV; some acknowledged the risk, while others did not. Participants reported inconsistent condom use. Some participants stated that they do not use condoms when having sexual intercourse with their regular partners, but they always use condoms when having sex with different partners. One participant believes that having one partner eliminates the risk of contracting HIV, whereas others stated that they consistently use a condom to reduce their chances of contracting HIV. Participants reported that:

‘Not at risk because I have one partner.’ (Participant 1, Male, 20 years old)

‘No, I use a condom every time I mess around but with my girlfriend, I do not use a condom, I trust her.’ (Participant 5, Male, 18 years old)

‘No, I use condoms during sex.’ (Participant 6, Female, 24 years old)

‘No, I protect myself with a condom, and I would use gloves when dealing with bloody situations and if it happens that I don’t have gloves with I would use plastics to help.’ (Participant 7, Male, 25 years old)

‘Yes, my girlfriend has twice cheated on me and she loves partying with friends on weekends, so I don’t know if she uses protection when she sleeps around though we sometimes don’t use it when I have with her.’ (Participant 14, Female, 20 years old)

‘Yes, my partner sometimes does not want to use a condom, but I always refuse sex if there is no condom.’ (Participant 19, Male, 21 years old)

‘Yes, I seldom use a condom because I have been with him for almost three years.’ (Participant 22, Female, 24 years old)

#### Sub-theme 2.2: Sexual risk understanding

Participants acknowledge that sexual danger exists. They have reported that unprotected sex and oral sex increase the risk of contracting HIV. Participants also stated that having unprotected sex with someone about whose status they are unaware increases their risk because you cannot diagnose a person by looking at them. Participants also stated that engaging in sexual intercourse with a partner who was picked at taverns while intoxicated with alcohol can increase the likelihood of not using a condom. Participants reported that:

‘Sleeping around, having sex without a condom.’ (Participant 3, Male, 25 years old)

‘Being ignorant of HIV transmission, hooking up for sex in a tavern [*one-night stand*] no condom.’ (Participant 4, Female, 20 years old)

‘Having many partners, drinking and ending up ‘*ulahla*’ [random sex with a stranger]. This usually happens in girls or women when they are too drunk, they lust for sexual contact.’ (Participant 10, Male, 25 years old)

‘*Nyama enyameni* [Skin to Skin] request by partners especially male partners, people who do not disclose their status and pretend all is well even during sex by not or refusing to use a condom.’ (Participant 28, Male, 21 years old)

‘Oral sex especially private part sucking.’ (Participant 30, Male, 21 years old)

#### Sub-theme 2.3 Factors associated with sexual risk behaviour

Some participants reported the fact that people lose control over their sexual desires and seek sex immediately when they are drunk as evidence for their claim that alcohol was the cause of the problems, giving rise to the names “*uyalahla*” and “*uwinwe*.” Some respondents stated that having multiple partners can affect their hazardous sexual behavior, while others said that engaging in relationships and sexual activity with men who are older than them increases one’s risk of contracting HIV. Peer pressure is one factor contributing to an increase in sexually risky behavior, according to other participants.

The findings are displayed below:

‘Drinking alcohol way over the limit is my major problem that is one of the reasons I sometimes forget to use protection during sex.’ (Participant 4, Female, 20 years old)

‘I tend to have more sex when I am drunk, and girls take advantage of me since I always have money on for buying alcohol.’ (Participant 10, Male, 25 years)

‘When I am drunk “*ndiyalahla*” [*I end engaging in random sex*] but I am trying to break the habit.’

‘Having more than one boyfriend can put me at risk.’ (Participant 11, Female, 18 years)

‘Maybe, If I was a teenager I would say “peer pressure” but since I am an adult I should say “Love is blind” [*being made to believe the person loves me*].’ (Participant 25, Female, 18 years)

### Theme 3: HIV prevention strategies

#### Sub-theme 3.1: HIV education awareness programmes

Participants expressed a strong desire for educational programmes in the community because they believe many of the youth have been given incorrect information, which has led them to engage in unscientific practices such as washing the virus away with Dettol. Educational programmes will empower the youth to make informed decisions, and the programmes should include infected ‘activists’ who can share their experiences with the youth. This will inspire and encourage young people to take better care of themselves. Participants reported that:

‘Just like in my school there should be HIV and substance abuse education in my community because there is youth which is not schooling that needs to be educated. The youth in my community especially those who are living with HIV so that they could guide and teach us to be more aware.’ (Participant 2, Female, 24 years old)

‘Motivating youth to use condoms and ways of avoiding HIV infection. These must be held at least twice a year. Most of us know HIV is here and it kills but little formal information is shared by professionals. Most of us we share wrong information amongst from each other like if you have unprotected sex you soon wash with Dettol mixed with little “*madubula*” to wash your private part so that the HIV does not stick in the private part. The infected youth or people must be involved. This will help in motivating and waring of HIV infection consequences.’ (Participant 6, Female, 24 years old)

‘There must be support group programs, HIV and AIDS awareness campaigns for all ages but mostly target youth. The youth should be the major part, most you are ignorant but these lousy comments like “*inyama enyameni*” is more fun and cannot stand plastic. They further say HIV is for people not dogs. Nurses and other HIV relevant health staff must be involved.’ (Participant 20, Male, 22 years old)

Education programmes should reach out to everyone in the community, including those involved in cultural practices, rather than just the youth. The participant stated that a family member became infected as a result of those he trusted in transitioning him to manhood. He became infected because a contaminated blade was used on multiple people during circumcision. The participant reported:

‘There must be programs on initiation education, many young boys get infected by the sharing of the circumcision blade, and my brother was a victim also young boys, circumcision leaders and parents.’ (Participant 25, Male, 19 years old)

The education and awareness programme should be age appropriate and address issues that young people face in the community. Because nurses are associated with illness, allied professions such as social workers should lead community education programmes instead of nurses:

‘Youth programs, educating according to ages on how to manage their lives. Department of Health and Social Development and not only the Department of Health. Youth always associate nurses with illness so social workers will serve best.’ (Participant 17, Female, 25 years old)

#### Sub-theme 3.2: Distribution of condom and enrolment on pre-exposure prophylaxis

Participants reported that condom distribution and enrolment in pre-exposure prophylaxis (PrEP) will help to reduce HIV infections among the youth. Condoms should be easily accessible, particularly in places where sex is initiated, such as taverns. The youths should not have to struggle to find condoms. Anyone over the age of 13 should be eligible to start taking PrEP to prevent further infections in the community. Condom distribution should not be halted because of the inclusion of PrEP. Participants reported that:

‘I would distribute condoms hence in my community there aren’t any available condoms, you have to go to the clinic to get one, I would place them in taverns where most of sex is initiated, knowing each other’s status in a relationship is also helpful.’ (Participant 3, Male, 25 years old)

‘How I wish we could have PrEP for preventing further infections. PrEP offers counselling and some sort of injection that prevent formation of HIV when someone is raped or condom breaks. It must start from thirteen years. Condom use must not stop also.’ (Participant 22, Female, 24 years old)

#### Sub-theme 3.3: HIV testing services

A participant emphasised the importance of ongoing HIV testing services. The participants agreed that there is a need to bring HIV testing to schools, but that the testing services should maintain confidentiality, as opposed to clinics, where they believe confidentiality is not maintained. The participant reported that:

‘There must be frequent at our school to do HIV testing and it will be better because there will be confidentiality unlike at clinics where you are known you are going to do HIV test since they isolate HIV testing Centre from other clinic services. They must involve clinic HIV practitioners or hospital nurses.’ (Participant 29, Male, 25 years old)

## Discussion

### Theme 1: HIV and AIDS knowledge and attitudes in youth

Participants were questioned regarding their knowledge of the distinctions between HIV and AIDS. The significant differences in participants’ knowledge of HIV indicate a gap in their understanding. When some participants claimed that HIV and AIDS were the same, it was inferred that they did not see any differences. According to Kapila et al. (2016), HIV is the human immunodeficiency virus that causes HIV infection, and AIDS is a condition in which the immune system progressively deteriorates, allowing life-threatening infections and malignancies to flourish. Closing such knowledge gaps is essential in order to reach the 95-95-95 targets. By 2030, the goal was to identify 95% of all HIV-positive people, treat 95% of those diagnosed with ART, and achieve viral suppression for 95% of those on treatment. It is crucial that youths have accurate information about HIV and AIDS accessible to them. Joint United Nations Programme on HIV/AIDS (UNAIDS 2018) emphasises that in order to reduce the number of HIV infections in the future, youths must be prioritised as the primary users of prevention approaches. The findings demonstrate that the participants understood the mode of transmission because they brought up sex and vertical transmission. According to Zuma et al. (2022), heterosexual partnerships are mostly responsible for HIV transmission in South Africa.

The participants were also questioned about the HIV transmission mechanism. As far as the majority of participants knew, tainted blood and cuts are the two most typical ways that HIV infections are spread. Barnett and Whiteside (2006) characterised HIV as a concentrated seminal fluid. The HIV≈can also be transmitted by blood products, patients who have received tainted blood transfusions, contaminated transplanted tissues, sharing infected needles or syringes among drug users, and needle sticks by occupational health professionals. Sharing sex toys without sterilising them and nursing from an HIV-positive mother are two more ways that HIV might spread (Adegoke 2010).

Questions addressing the participants’ opinions of HIV and AIDS were posed. The participants’ responses make it clear how important it is to start treatment as soon as an HIV positive test result is obtained. While the majority of participants did not agree that seeking treatment from a traditional healer is necessary once a person develops sores in their private area, some did report that HIV is curable and that public acceptance of the virus could help an individual lead a normal life.

The authors questioned the participants about how they felt using condoms. The vast majority of participants also discussed their varied opinions regarding condom use. This has changed their perspective on condom use. Some participants think that by reducing sexual sensation, condom use decreases the pleasure of having sex. However, other individuals admitted that they hardly ever used condoms during sexual encounters. The participants went on to say that because condom use can help prevent HIV, STIs, and unwanted pregnancies, it is vital. The participants also mentioned that condoms can prevent HIV from spreading, but they also expressed dissatisfaction with them because they burst during sexual activity. The results of the study demonstrate that young people value using condoms to avoid getting HIV, getting pregnant, and getting other STIs. According to Shisana, Rehle and Simbayi (2014), regular condom users have a 20-fold lower risk of HIV infection than irregular users. Recent data from the South African Demographic and Health Survey show that 50% of young women and 66% of young men reported having sex before the age of 18, and only 72.9% and 62.3%, respectively, reported using a condom (Davids et al. 2021). Hamid and Associates (2022) indicated that poor condom use has been linked to a number of factors, including decreases sexual satisfaction, difficulty using, feeling shy to buy condoms, difficulty persuading a partner to use them, the cost of condoms, aversion to the condom, drinking or using drugs prior to sexual activity, a lack of knowledge and skills in a sexual relationships, social stigma, and moral and religious considerations.

### Theme 2: Sexual risk behaviour among youth

Participants’ perceptions of sexually risk behaviour were asked. The participants discussed a range of STIs. The following were among the responses from the participants: oral sex, having multiple sexual partners, drinking excessively and losing self-control, having sex without a condom, not knowing one’s HIV status, and having sex with older partners. It is believed that several sexual partners contribute to the transmission of STIs, such as HIV (USAID 2013). Maughan-Brown (2013) states that people who have multiple sexual partners are believed to be more susceptible to HIV infection. People who have had several partners in unprotected sexual activity may be more susceptible to HIV infection (Southern Africa HIV and AIDS Information Dissemination Services 2014).

When asked how they could be at risk of HIV infection, the participants admitted to engaging in high-risk sexual behaviour. High-risk behaviours include not using a condom during sexual activity, having multiple partners, using a condom inconsistently, engaging in unsafe oral sex, being afraid of testing, and having sex while intoxicated. People who have multiple partners and engage in unprotected sexual activity may be more susceptible to HIV infection (Southern Africa HIV and AIDS Information Dissemination Services 2014). Men are believed to be more likely than women to be in multiple partnerships at the same time (Onoya et al. 2014). Furthermore, Tadesse and Yakob (2015) assert that if young people do poorly and want attention from men and their peers, they are more likely to engage in risk sexual practices.

Unawareness of the risks associated with unprotected sex increases a young person’s risk of contracting STDs, becoming pregnant, or impregnating someone else. According to Tadesse and Yakob (2015), a lack of understanding and basic skills for emotion management may also contribute to this behaviour, as well as strong peer pressure to experiment sexually. Drug and alcohol abuse are known to increase the risk of HIV transmission, both directly and indirectly. This occurs indirectly because substances such as alcohol can lower inhibitions and impair judgement. As a result, there is an impact on risky sexual behaviors such increasing the number of sexual partners and experiences (Parry, Carney & Petersen Williams 2017).

### Theme 3: HIV prevention strategies

According to Chetty-Makkan et al. (2020) Human Immunodeficiency Virus infection is having a devastating effect on youth, and it is no longer acceptable to overlook its effects. Without a doubt, HIV infection primarily affects young people (Chetty-Makkan et al. 2020). Participants acknowledge young people’s exposure to HIV infection. They disagree on whether they are in danger or not, which has caused division among them. The split may be to blame for the low rate of HIV testing among youth. According to Chetty Makkan and colleagues (2020), young people’s lack of HIV testing may be caused by the division. According to these authors, a few reasons why young people do not test for HIV include their lack of knowledge about the virus, their fear of having their privacy violated or receiving a positive diagnosis, the stigma associated with the condition, and the requirement for consent from parents or guardians.

The results of the study indicate that the implementation of prevention programmes will lower the rate of HIV infection among youth, which will impact the 95-95-95 objective. Programmes for HIV awareness, condom distribution, PrEP enrollment, and HIV testing services have all been mentioned by participants as potentially reducing HIV among young people in the community.

Awareness-raising efforts are still a potent means of connecting with people. Participation in educational or media programmes might help raise awareness. The significance of leveraging and strengthening awareness programmes has been emphasised by participants. They have made hints that educational awareness campaigns can help debunk the widespread misunderstanding that young people face. Understanding HIV and STIs is necessary to improve young people’s abilities to practise safe sex and to reduce unfavourable attitudes towards condom use (Khamisa et al. 2020).

Participants have stated that they would like condoms to be readily available in their local areas. They’ve said that youths engage in sexual activity while inebriated and that condom availability in bars and other public settings, will lower the rate of HIV infections. It has been claimed that accurate and consistent condom usage reduces the risk of HIV infection during sexual activity in heterosexual couples by 70%, regardless of the individual’s knowledge of their status or level of ART compliance. The foundation of public health HIV prevention initiatives for a long time has been condom supply interventions (Malekinejad et al. 2017). It is imperative that information be aimed at youth and that condom promotion continue.

The PrEP is a biological approach that offers extra HIV prevention choices to at-risk persons beyond standard programmes such as condom use. A daily tablet called PrEP is given to prevent HIV infection (Walters et al. 2017). According to Lefoka and Netangaheni (2021), treating an infected individual with antiretroviral medication for the remainder of their life would be far more expensive than the cost of PrEP. The PrEP for youth could reduce the HIV epidemic and save the government a significant amount of money.

The HIV preventive services can be accessed through Human Immunodeficiency Virus Counselling and Testing (HCT), which is the first in a sequence of interventions for HIV prevention and care (Lefoka & Netangaheni 2021). Men, the elderly, and teenagers in South Africa are less likely to use HCT (Mabuto et al. 2014). The participants found that they needed to have access to HIV testing services, and they also mentioned that these services should be offered in a school setting, where students might SMS one another. According to a study conducted by Carnevale et al. (2022), young people who live in communities with some of the highest rates of HIV seroprevalence in the country and have access to confidential sex-positive healthcare within their schools, are more likely to seek out PrEP and other HIV services. This finding is consistent with what the researchers found. In HIV preventive measures for youth, HCT should not be disregarded (Lefoka & Netangaheni 2021).

### Strengths and limitations

The study’s strength is that, in the light of HIV and AIDS trends, young people in rural areas, particularly those in deep rural areas, are frequently ignored. They are frequently left with prior knowledge, which they occasionally have a tendency to dismiss as unstable.

The study research limitations are as follows: Only individuals in the age range of 18 years to 25 years took part in the study, with 30 young people from Nyandeni Municipality making up the sample. Since a qualitative research design was used in the study, it is not possible to generalise the findings to all young people. Even though the Nyandeni Municipality has seen a number of research studies on attitudes and understanding related sexual risk behaviours, this is the first to target young people between the ages of 18 years and 25 years particularly. Selection bias may have resulted from the purposive sampling technique used to pick study participants in non-probability studies. Because of the inherent bias of face-to-face interviews, it is feasible that certain participants will give responses that are acceptable in society. The absence of data saturation may suggest that the study sample was insufficient for the topic under investigation.

## Recommendations

### Recommendation based on the findings of the research

- Intense awareness programmes are required to educate youth about the risks associated with risk sexual behaviour. Both adolescents who have dropped out of school and youth who are currently enrolled should be included in the new tactics.
- The appropriate authorities should frequently update advanced adolescents who believe they lack sufficient understanding about HIV and AIDS during informational workshops so that they are accurately informed about this illness. Youths should also be informed of any updates to HIV and AIDS incidence figures. This ought to cover the modifications in the virus’s mutation. Programme administrators need to be cautious about assuming that information will inevitably lead to desired behaviours.
- It is imperative that youths have access to accurate and lucid information on HIV and AIDS from all accessible sources. Information about using condoms effectively must be included in this. It is necessary to check periodicals, newspapers, radio, and television shows to ensure that the information they give is accurate and unadulterated. It’s important to wear condoms appropriately; certain condoms have instructions on how to accomplish this.

### Recommendation based on future research

- The participants in this study were exclusively young people aged 18 to 25. The researcher would like to suggest that a study similar to this one be carried out in young people between the ages of 13 and 35. Because the majority of young people start having sexual relations at age 13, any study that looks at young people over 25–35 years old should include them.

### Recommendation based on policy and practice

- Law enforcement agents should be strengthened and clamp down on people who sell drugs, stop taverns from opening until the wee hours of the morning, and stop organising sex parties in the community in order to curb the exposure of young people, especially those under the age of 18, even though they were not part of the study.
- The establishment of a life skills centre in the communities of Nyandeni Municipality is recommended. The two NPOs that were part of this study are not enough to deliver the necessary services because of demarcation, and many youth in the Municipality cannot access them. Most youths did not even know that there were such NPOs in their Municipality.

## Conclusion

The main conclusions of this study highlight the knowledge and attitudes related to HIV and sexual risk behaviours among youth aged 18–25 years in the Nyandeni Municipality in the Eastern Cape province. It also revealed that some participants were placing themselves at risk of HIV infection because they knew very little about HIV and AIDS. This exploratory investigation supports the conclusion that the participants’ knowledge is limited by demonstrating that the majority of them knew very little about HIV and AIDS infection and prevention. Ongoing educational initiatives are necessary. One potential way to achieve this goal is through the community’s proposed adoption of several educational initiatives. This survey showed that the usage of condoms was common, and it has to be handled with the same fervour. It is important to teach and instruct young people on the usage of condoms and the problems that can occur from using them. Additionally, PrEP can help prevent HIV, hence more PrEP services have to be provided.

## References

[CIT0001] Abdullah, F., Naledi, T., Nettleship, E., Davids, E.L., Van Leeuw, L., Shangase, S.L. et al., 2019, ‘First social impact bond for the SAMRC: A novel financing strategy to address the health and social challenges facing adolescent girls and young women in South Africa’, *South African Medical Journal* 109(11b), 57. 10.7196/samj.2019.v109i11b.14254 32252870

[CIT0002] Adegoke, S.O., 2010, *Effect of HIV/AIDS awareness on sexual behaviour of students in tertiary institutions in Nigeria*, University press, Ilorin.

[CIT0003] Barnett, T. & Whiteside, A., 2006, *AIDS in the 21st century: Disease and globalizatio n*, Palgrave Macmillan, New York, NY.

[CIT0004] Bossonario, P.A., Ferreira, M.R.L., Andrade, R.L., De, P., Sousa, K.D.L., De Bonfim, R.O. et al., 2022, ‘Risk factors for HIV infection among adolescents and the youth: A systematic review’, *Revista Latino-Americana de Enfermagem* 30(spe), e3695. 10.1590/1518-8345.6264.3695 PMC9647917 36197391

[CIT0005] Carnevale, C., Zucker, J., LaSota, E., Northland, B., Arache, A., Peralta, H. et al., 2022, ‘PreexposureProphylaxis at School-Based Health Centers: Awareness and interest in starting Preexposure Prophylaxis while attending a School-Based Health Center in New York City’, *Journal of Pediatric Health Care* 36(3), 256–263. 10.1016/j.pedhc.2021.10.006 34840056

[CIT0006] Chetty-Makkan, C.M., Hoffmann, C.J., Charalambous, S., Botha, C., Ntshuntshe, S., Nkosi, N. et al., 2020, ‘Youth preferences for HIV testing in South Africa: Findings from the Youth Action for Health (YA4H) study using a discrete choice experiment’, *AIDS and Behavior* 25(1), 182–190. 10.1007/s10461-020-02960-9 32607914

[CIT0007] Creswell, J.D. & Creswell, J.W., 2018, *Research design – Qualitative, quantitative, and mixed methods approaches*, 5th illustrated edn., SAGE Publications, Los Angeles, CA.

[CIT0008] Davids, E.L., Zembe, Y., De Vries, P.J., Mathews, C. & Swartz, A., 2021, ‘Exploring condom use decision-making among adolescents: The synergistic role of affective and rational processes’, *BMC Public Health* 21(1), 1894. 10.1186/s12889-021-11926-y 34666719 PMC8527692

[CIT0009] Forero, R., Nahidi, S. & De Costa, J., 2018, ‘Series: Practical guidance to qualitative research Part 4: Trustworthiness and publishing’, *European Journal of General Practice* 24(1), 120–124. 10.1080/13814788.2017.1375092 29202616 PMC8816392

[CIT0010] Gray, J.R., Grove, S.K. & Sutherland, S., 2020, *Burns & Grove’s the practice of nursing research: Appraisal, synthesis, and generation of evidence*, 9th edn., Elsevier, St. Louis, MO.

[CIT0011] Hamid, NC., Malek, K.A., Mat-Nasir, N., Mohamad, M. & Nasir, N.M., 2022, ‘Prevalence of good condom usage and its association with condom use self-efficacy among youth attending HIV/STDs clinics in primary-care settings in Malaysia’, *International Journal of Environmental Research and Public Health* 19(19), 12179. 10.3390/ijerph191912179 36231478 PMC9565083

[CIT0012] Jense, E. & Laurie, C., 2016, *Doing real research: A practical guide to social research*, Sage, Thousand Oaks, CA.

[CIT0013] Kapila, A., Chaudhary, S., Vashist, H., Sisodia, S.S. & Gupta, A., 2016, ‘A review on: HIV AIDS’, *Indian Journal of Pharmaceutical and Biological Research* 4(03), 69–73. 10.30750/ijpbr.4.3.9

[CIT0014] Khamisa, N., Mokgobi, M. & Basera, T., 2020, ‘Knowledge, attitudes and behaviours towards people with HIV and AIDS among private higher education students in Johannesburg, South Africa’, *Southern African Journal of HIV Medicine* 21(1), a991. 10.4102/sajhivmed.v21i1.991 PMC7136969 32284887

[CIT0015] Korstjens, I. & Moser, A. 2018, ‘Series: Practical guidance to qualitative research. Part 4: Trustworthiness and publishing’, *European Journal of General Practice* 24(1), 120–124. 10.1080/13814788.2017.1375092 29202616 PMC8816392

[CIT0016] Kumar, R., 2018, *Research methodology: A step-by-step guide for beginners*, Sage, London. 10.7748/nr.19.3.45.s5 27712357

[CIT0017] Kyngäs, H., Kääriäinen, M. & Elo, S., 2019, ‘The trustworthiness of content analysis’, in *The application of content analysis in nursing science research*, pp. 41–48.

[CIT0018] Lefoka, M.H. & Netangaheni, R.T., 2022, ‘Factors associated with smoking and transitioning to Nyaope injection among women in the City of Tshwane Municipality: A self-report by women’, *Health SA Gesondheid* 27, a1775. 10.4102/hsag.v27i0.1775 PMC9350489 35937427

[CIT0019] Lima, M.S., De Raniere, J.C., Paes, C.J.O., Gonçalves, L.H.T., Cunha, C.L.F., Ferreira, G.R.O.N. et al., 2020, ‘The association between knowledge about HIV and risk factors in young Amazon people’, *Revista Brasileira de Enfermagem* 73(5), e20190453. 10.1590/0034-7167-2019-0453 32667402

[CIT0020] Mabuto, T., Latka, M.H., Kuwane, B., Churchyard, G.J., Charalambou, S. & Hoffmann, C.J., 2014, ‘Four models of HIV counseling and testing: Utilization and test results in South Africa’, *PLoS One* 9(7), e102267. 10.1371/journal.pone.0102267 25013938 PMC4094499

[CIT0021] Malekinejad, M., Parriott, A., Blodgett, J.C., Horvath, H., Shrestha, R.K., Hutchinson, A.B. et al., 2017, ‘Effectiveness of community-based condom distribution interventions to prevent HIV in the United States: A systematic review and meta-analysis’, *PLoS One* 12(8), e0180718. 10.1371/journal.pone.0180718 28771484 PMC5542551

[CIT0022] Maughan-Brown, B., 2013, ‘Concurrent sexual partnerships among young adults in Cape Town, South Africa: How is concurrency changing?’, *Sexual Health* 10(3), 246. 10.1071/SH12148 23680089

[CIT0023] Mullick, S., 2023, ‘Sexually transmitted infections in pregnancy: Prevalence, impact on pregnancy outcomes, and approach to treatment in developing countries’, *Sexually Transmitt ed Infections* 81(4), 294–302. 10.1136/sti.2002.004077 PMC1745010 16061534

[CIT0024] Nieswiadomy, R.M. & Bailey, C., 2018, *Foundations of nursing research*, 7th edn., Pearson, New York, NY.

[CIT0025] Nyandeni Local Municipality (NLM), 2018, *Nyandeni local municipality final integrated development plan (IDP) review 2018–2019*, Nyandeni Local Municipality, Libode, viewed 29 August 2023, from https://www.nyandenilm.gov.za/storage/wp-content/uploads/2019/08/Final-Nyandeni-LM-LED-Strategy-Review-pdf.pdf.

[CIT0026] Onoya, D., Zuma, K., Zungu, N., Shisana, O. & Mehlomakhulu, V., 2014, ‘Determinants of multiple sexual partnerships in South Africa’, *Journal of Public Health* 37(1), 97–106. 10.1093/pubmed/fdu010 24639477

[CIT0027] Parry, C.D.H., Carney, T. & Petersen Williams, P., 2017, ‘Reducing substance use and risky sexual behaviour among drug users in Durban, South Africa: Assessing the impact of community-level risk-reduction interventions’, *SAHARA-J: Journal of Social Aspects of HIV/AIDS* 14(1), 110–117. 10.1080/17290376.2017.1381640 PMC5639608 28969490

[CIT0028] Pleaner, M., Scorgie, F., Martin, C., Butler, V., Muhwava, L., Mojapele, M. et al., 2023, ‘Introduction and integration of PrEP and sexual and reproductive health services for young people: Health provider perspectives from South Africa’, *Frontiers in Reproductive Health* 4, 1086558. 10.3389/frph.2022.1086558 36699145 PMC9869154

[CIT0029] Tadess, G. & Yakob, B., 2015, ‘Risk sexual behaviours among female youth in Tiss Abay. A semi-urban area of the Amhara Region, Ethiopia’, *PLoS One* 10(3), 0119050. 10.1371/journal.pone.0119050 PMC4349819 25738508

[CIT0030] Shamu, S., Khupakonke, S., Farirai, T., Slabbert, J., Chidarikire, T., Guloba, G. et al., 2020, ‘Knowledge, attitudes and practices of young adults towards HIV prevention: An analysis of baseline data from a community-based HIV prevention intervention study in two high HIV burden districts, South Africa’, *BMC Public Health* 20(1), 1249. 10.1186/s12889-020-09356-3 32807116 PMC7433171

[CIT0031] Shisana, O., Rehle, T. & Simbayi, L., 2014, *South African National HIV prevalence, incidence and behaviour survey*, HSRC Press, Cape Town. 10.1080/09540121.2015.1080790 PMC5146982 26551532

[CIT0032] Southern Africa HIV and AIDS Information Dissemination Service (SA HAIDS), 2014, *Multiple and concurrent partnerships: Driving Southern Africa’s HIV epidemic*, viewed 29 August 2023, from http://www.safaids.net/files/MCPHowtocard4.Pdf.

[CIT0033] Statistics SA, 2023, ‘Mid-year population estimates 2022’, in Statistics South Africa (ed.), *Statistical release P0302*, Statistics South Africa, Pretoria.

[CIT0034] UNAIDS, 2018, *UNAIDS data*, Geneva, viewed 20 August 2023, from http://www.unaids.org/sites/default/files/mediaasset/unaids-data-2018_en.pdf/.

[CIT0035] USAID, 2013, *U.S. Congress reauthorized the U.S. President’s emergency plan for AIDS relief (PEPFAR)*, Tetra Tech ARD.

[CIT0036] Varshney, K., Browning, S.D., Debnath, S.K., Shet, P. & Shet, D., 2022, ‘A systematic review of risk factors and consequences of nyaope usage: The illicit street drug containing HIV antiretrovirals’, *AIDS Behavior* 27(2), 558–577. 10.1007/s10461-022-03791-6 35895149 PMC9908705

[CIT0037] Walters, S.M., Reilly, K.H., Neaigus, A. & Braunstein, S., 2020, ‘Awareness of pre-exposure prophylaxis (PrEP) among women who inject drugs in NYC: The importance of networks and syringe exchange programs for HIV prevention’, *Harm Reduction Journal* 14(1), 40. 10.1186/s12954-017-0166-x PMC5492910 28662716

[CIT0038] Woollett, N., Pahad, S. & Black, V., 2021, ‘“We need our own clinics”: Adolescents’ living with HIV recommendations for a responsive health system’, *PLoS One* 16(7), e0253984. 10.1371/journal.pone.0253984 34197529 PMC8248739

[CIT0039] Zuma, K., Simbayi, L., Zungu, N., Moyo, S., Marinda, E., Jooste, S. et al., 2022, ‘The HIV epidemic in South Africa: Key findings from 2017 National Population-Based Survey’, *International Journal of Environmental Research and Public Health* 19(13), 8125. 10.3390/ijerph19138125 35805784 PMC9265818

